# Substantial Variation Exists in Clinical Exposure to Chief Complaints Among Residents Within an Emergency Medicine Training Program

**DOI:** 10.5811/westjem.20281

**Published:** 2024-11-19

**Authors:** Corlin M. Jewell, Amy T. Hummel, Dann J. Hekman, Benjamin H. Schnapp

**Affiliations:** * University of Wisconsin School of Medicine and Public Health, BerbeeWalsh Department of Emergency Medicine, Madison, Wisconsin; † Emergency Medicine Specialists SC, Wauwatosa, Wisconsin

## Abstract

**Introduction:**

While many aspects of emergency medicine (EM) residency training are standardized among residents within a single residency program, there is no standard for the distribution of chief complaints (CC) that residents should see over the course of residency. This could result in substantial variability in each resident’s clinical exposure. Our objective in this study was to explore EM residents’ clinical exposure to CCs to determine whether substantial variation exists. If such variation exists, this could suggest the need for curricular reform to address gaps in resident clinical exposure during training.

**Methods:**

This was a retrospective observational study of EM residents who graduated in the years 2016–2021 at a single, university-affiliated emergency department (ED) in the midwestern United States. All patient encounters where a CC was logged were included and categorized into 1 of 20 clinical domains based on the 2016 American Board of Emergency Medicine Model of Clinical Practice. We calculated descriptive statistics for the top 10 most encountered domains for comparison among residents.

**Results:**

We included a total of 228,916 patient encounters from 69 residents in the analysis. Residents were involved in an average of 3,323 distinct patient encounters during the study period. The overall interquartile range for patient encounters was 523. The three CC domains with the broadest interquartile variation were abdominal and gastrointestinal disorders (116), musculoskeletal disorders (nontraumatic) (93), and traumatic disorders (86).

**Conclusion:**

Within a single, three-year academic EM program, substantial variation existed among residents with regard to the variety of patient CCs seen during their residency training.

Population Health Research Capsule What do we already know about this issue?

*Studies from 30 years ago reported variation in the distribution of chief complaints seen by emergency medicine residents during training.*

What was the research question?

*We hypothesized that substantial differences in clinical exposure still exist among residents at the time of graduation.*

What was the major finding of the study?

*The three chief complaint domains with the most variability between individual resident experience, as measured by the greatest 25–75 interquartile ranges were abdominal and gastrointestinal disorders (median 594 patients per resident, IQR 116), nontraumatic musculoskeletal disorders (median 314, IQR 92), and traumatic disorders (median 525, IQR 86).*

How does this improve population health?

*Understanding these differences is important, as substantial variation could mean that some residents do not develop robust illness scripts suitable for independent practice.*

## INTRODUCTION

Medical residency training allows physicians to gain the cognitive and procedural skills necessary to practice independently. Based on experiential learning theory, patient encounters form the foundation upon which physicians in training begin to master the practice of medicine.^1^ Additionally, the development of “illness scripts,” or mental models for the classification of patient presentations, is crucial to the development of clinical skills and reasoning during residency training.^2^ These models are developed over time by multiple exposures to presentations of similar disease states.^3^ ^,^ ^4^ Emergency medicine (EM) trainees must be exposed to a variety of patient chief complaints (CC) throughout the course of residency to develop these scripts and become ready to begin independent practice.

Educators within EM have worked to define many aspects of EM residency training, including optimum number of shifts, on-shift educational goals/practices, and didactic content.^5^ Despite this, the clinical experience of an individual resident may be highly variable and may be partially driven by self-selection of patients by the resident. Studies in pediatric EM suggest that there is significant variation in the overall number of patients and range in acuity among individual residents.^6^ ^,^ ^7^ However, there is little adult EM literature that explores the variation in clinical experience seen by residents within a modern EM program. The literature that does exist in adult EM suggests there is substantial variation in clinical exposures among residents.^8^ A study from 2006 found that the number of cases seen overall correlated with improved performance on a standardized test designed to assess clinical competence. However, the effect plateaued at around 200 cases.^9^ Prior work by our group has shown that case volume in an individual domain did not correspond to performance within that domain on corresponding questions on the in-training exam.^10^

These studies suggest that individuals within a single training program may be gaining variable experience with certain types of patient presentations and lacking exposure (and therefore opportunities to develop mastery) to other complaints and pathology. However, this variability in clinical exposure during training has not been shown in adult EM for over three decades.^8^ Since then, the number of annual visits to the ED as well as the complexity of medical care provided have substantially increased.^11^ ^,^ ^12^ We, therefore, hypothesized that substantial differences in clinical exposure still exist among residents at the time of graduation. Understanding these differences is of critical importance for residency programs as considerable variation could push some residents below a threshold to develop robust illness scripts suitable for independent practice.

## METHODS

### Study Design and Setting

We conducted this retrospective, observational study at a three-year EM residency program situated within an urban, academic emergency department (ED) in the Midwest. The ED for the primary clinical site has a total of 54 beds and sees an annual volume of approximately 60,000 patient visits. During the study period, the residency had 12 first postgraduate year one (PGY-1) positions available each year. The study ED divides its beds into two adult clinical areas and a pediatric clinical area. All three areas are physically connected on a single floor of the hospital. Residents from all three years are assigned to nine-hour shifts in each clinical area. Each shift includes 1–2 junior (PGY-1) residents, 1–2 senior (PGY-2 or PGY-3) residents, and one attending physician. Any resident can assign themselves to patients of any severity regardless of seniority. In Fall 2020, the study ED shifted from a “pod” model in which the two adult clinical areas would assign themselves predominately to patients in their clinical area to a “free-for-all” model in which either adult team could assign themselves to any adult patient regardless of the clinical area they were roomed in. During the study, physician assistants were employed in the ED and would occasionally take the place of a resident on shift (particularly during weekly resident didactics).

### Data Acquisition

Residents were eligible for inclusion if they had completed residency within three consecutive years and graduated in the years 2016–2021 (therefore, the study period was from June 2013–June 2021). The electronic health record (EHR) was used to create a database of patient encounters; all encounters where eligible EM residents were the first resident assigned to the patient were analyzed. We used deidentified patient encounter data, listed by first CC. The CC was used to identify the nature of the patient encounter as this data was available at the time of patient presentation, often dictates the patient’s ED workup, and would not have been affected by information discovered during the later stages of a patient’s hospital course. This approach is consistent with prior literature.^9^ ^,^ ^13^ To maintain anonymity, only the senior author, a member of the residency leadership team, had access to each resident’s individualized study identification number.

We excluded from analysis encounters where no CC was listed or no resident was assigned. In cases where multiple residents were assigned to a single encounter (e.g., a patient had been signed out to a different resident), we analyzed this encounter only for the initial resident assigned. This was done as they are typically the most involved in the cognitive workload of determining the patient’s initial diagnostic and treatment plan. The CC for each encounter was selected and entered into the EHR by the primary nurse who cared for the patient in the ED initially. At our institution, this is nearly always selected from a list of common CCs, although it can be entered as free text. Encounters in which multiple CCs were listed were only coded into a single domain based on the first listed CC.

### Data Analysis

A list of common CCs in EM has been categorized into a set of 20 content domains via a consensus process by two EM attendings using the 2016 American Board of Emergency Medicine (ABEM) Model of Clinical Practice as a framework.^14^ For CCs identified in our data that were not already categorized by a previously described method,^13^ we repeated the same categorization process in which each CC was assigned to a single domain by two board-certified EM attending physicians at our institution. Disagreements between the two reviewers were adjudicated by a third board-certified emergency physician. If a symptom was entered as the CC, such as “fever” (which could correspond to one of multiple domains), it was preferentially categorized into a domain based on what the coding physicians felt was the most likely to dictate the ED workup, rather than the “signs, symptoms, and presentations” domain. We used Excel (Microsoft Corp, Redmond, WA) to calculate descriptive statistics and create plots and tables. The top 10 most encountered domains overall were analyzed. We excluded less common domains given the low number of total encounters in each area, which would have been more vulnerable to random fluctuations in when these patients present to the ED.

This project was deemed exempt quality improvement by the University of Wisconsin Health Sciences Institutional Review Board.

## RESULTS

A total of 315,614 encounters were initially identified from the EHR. Of these encounters 198 were excluded as no CC was listed. After excluding residents whose clinical experience was outside the study period and those who had left the training program prior to graduation or had a prolonged leave of absence, a total of 228,916 patient encounters from 69 residents were included in the analysis. Each resident was assigned to an average of 3,323 distinct patient encounters Assessment of the top 10 most common clinical exposure domains showed wide ranges in the case numbers of individual residents. The Table lists the mean, minimum, maximum, interquartile range (IQR) and 25th and 75th percentile for the 10 most common content domains. The Figure shows the range of exposure to the 10 most common domains in box-and-whisker format.

**Table.** Mean, 25th–75th percentile ranges, interquartile range, and minimum/maximum encounters for the 10 most encountered domains per resident.

	Mean	Median	25^th^, 75^th^ percentile	IQR	Minimum, maximum
Total encounters	3323		3086, 3609	523	2595, 4053
Abdominal and gastrointestinal disorders	583	594	528, 644	116	416, 721
Traumatic disorders	529	525	484, 570	86	370, 725
Cardiovascular disorders	327	330	302, 356	54	233, 429
Nervous system disorders	319	319	301, 340	39	226, 402
Musculoskeletal disorders (non-traumatic)	314	314	269, 361	92	179, 460
Thoracic-respiratory disorders	280	281	246, 313	67	178, 383
Systemic infectious disorders	165	169	149, 179	30	115, 219
Head, ear, eye, nose, and throat disorders	150	151	136, 165	29	96, 196
Signs, symptoms, and presentations	129	130	120, 142	22	88, 170
Psycho-behavioral disorders	126	128	106, 139	34	67, 211

*IQR*, interquartile range.

**Figure.**
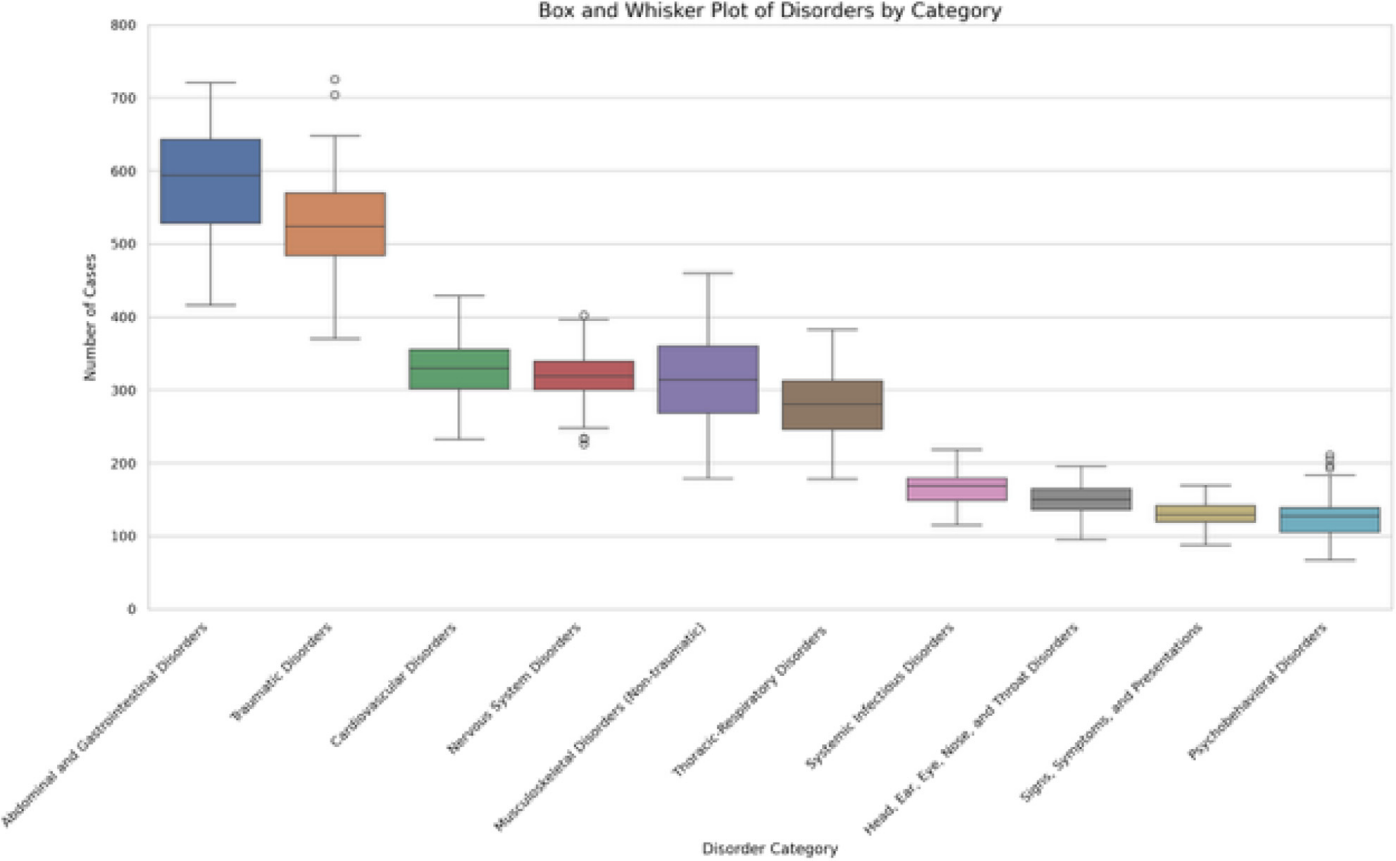
Top 10 most common clinical exposure domains seen by graduation per resident. Boxes illustrate the 25th–75th percentile of number of clinical exposures by residents in each domain, with whiskers representing the minima, maxima, and outliers.

## DISCUSSION

Our data suggests that residents within a single training program have substantial variation in their clinical experiences as measured by the variation in ABEM content domains seen by individual residents. This is similar to what was described by Langdorf et al. in 1990, despite the previous study being performed over three decades ago and the substantial subsequent differences in the utilization of the ED.^8^ We found wide interquartile ranges between the maximum and minimum number of encounters among residents, suggesting that some residents saw substantially more patients within particular domains than others.

The magnitude of the educational significance of the exposure variability of residents is unclear. It is possible that a resident who sees twice as many musculoskeletal chief complaints as another resident by graduation is significantly more competent in that domain. Alternatively, it is also possible that they have both attained the minimal level of exposure to competently manage musculoskeletal complaints independently. The effects of clinical exposure on clinical competence, including the minimal number of encounters required to demonstrate competency in a particular domain, is an open question and an avenue for further research. However, the formation of illness scripts is continually modified by subsequent patient encounters.^3^ ^,^ ^4^ Therefore, the identification of high degrees of variation among residents may prompt program leadership to institute changes in the curriculum or supplement clinical exposure with individualized learning plans. This is likely more important for domains that are encountered less frequently overall, such as psycho-behavioral disorders, where larger relative differences in exposure could result in greater deficits in illness script formation.

In addition to prompting changes made by the program, identification of high variability in clinical exposure may enhance resident self-assessment. As demonstrated previously, self-assessment when done in isolation, is an imperfect means of driving improvement but can be enhanced greatly when informed by additional information from a variety of sources.^15^ Understanding the distribution of the patient encounters residents have during training, and the potential gaps in their clinical exposure, could be a potential means of allowing for informed self-assessment for a resident’s clinical skills. This could be potentially further enhanced if facilitated under the supervision of faculty coaches within the program, a method that has become increasingly popular in medical education.^16^ ^,^ ^17^ Future work could follow a cohort of residents who are able to track their own patient volumes more regularly than was possible in the current study and compare themselves to their peers throughout training and evaluate whether any differences in clinical competence are identified. This could also allow programs to determine the perceived value of this information to residents. Finally, residents could use this data to drive their patient selection while working in the ED.

Beyond the potential for shaping resident self-assessments, clinical exposure data may have important implications for residency program leadership as we move toward an era of competency-based medical education (CBME). Two of the pillars of CBME, “teaching tailored to competencies” and “effective programmatic assessment,”^18^ lend themselves well to the identification of program clinical weaknesses as well as to the creation of new curricular experiences designed to address areas of limited clinical exposure identified by resident CC data. These experiences could potentially take the form of targeted readings or simulation sessions designed to supplement lower frequency clinical encounters.

## LIMITATIONS

This was a single-center study in an urban, academic ED, and findings may not be generalizable to training programs in different environments. Additionally, the data was retrospective, making the educational utility of this information or any potential causes of variation difficult to determine.

Use of a CC to categorize each patient encounter into a clinical domain has an element of subjectivity and may have led to some encounters being miscategorized with respect to the workup done or final diagnosis. Some additional subjectivity may have been introduced by how we classified CCs that could potentially have been categorized into multiple different domains (such as “fever” or “ingestion”). This was done based on what was determined to be most likely to drive the initial workup in the department. For example, although a CC of “chest pain” could represent a cardiac or pulmonary etiology, in almost all cases, a cardiac etiology must be excluded. Therefore, it was felt that this would influence the formation and modification of the resident’s illness script most heavily. It is also possible that encounters were mischaracterized due to only using the first CC listed and not considering the others if multiple CCs were listed. Like the prior limitation, it was felt that the first CC was most likely to dictate the initial ED workup. Using discharge or final diagnoses instead was considered for this study, but it was felt that the CC is more likely to drive the initial differential and diagnostic workup for the patient.

Additionally, ABEM domains may be too broad to capture important differences in exposure (e.g., two residents with the same exposure to “respiratory disorders” could have seen large numbers of pneumonia patients or, alternatively, many patients with asthma). Training is inherently variable as the EM environment differs by clinical site, day, shift, or even season. Therefore, there may have been slight differences in when individual residents were in the ED clinically or the number/type of overall ED shifts worked. It is important to note that some of the included residents’ training occurred during the COVID-19 pandemic, which may have had an effect on both the variety and number of clinical exposures seen by these residents. Future work could also explore exposure based on sub-domains from the ABEM model to get a more granular look at individual resident clinical experiences rather than relying on the relatively broad domains.

Other clinical variables may also have an effect on a resident’s clinical exposure, including the timing of months rotating in the ED. However, the ED did not undergo major changes in the staffing model of physicians (including residents) during this period. Also, while it is likely that more senior residents assign themselves to critically ill patients, this was felt to be unlikely to meaningfully impact our results given that data was obtained at the time of graduation. Therefore, each resident would have acted in a senior role for the same amount of time. Finally, our use of the EHR at the main clinical training site of the residency to generate the data did not capture the clinical experience at two other training sites for the residency that use a different EHR. This may have served to moderate or exacerbate the differences seen among residents. However, clinical experiences at these other sites comprised a total of only four months of the 36-month curriculum, and so it is likely that our overall findings would not have been substantially affected.

## CONCLUSION

Within a single, three-year academic emergency medicine program, there was substantial variation among residents regarding the variety of patient chief complaints seen throughout residency when mapped to ABEM’s Model of Clinical Practice.
